# IL-1β, the first piece to the puzzle of sepsis-related cognitive impairment?

**DOI:** 10.3389/fnins.2024.1370406

**Published:** 2024-04-11

**Authors:** Qing Zhu, Li Wan, Han Huang, Zhimin Liao

**Affiliations:** ^1^Department of Anesthesiology, Key Laboratory of Birth Defects and Related Diseases of Women and Children, West China Second University Hospital, Sichuan University, Chengdu, China; ^2^Department of Medical Genetics/Prenatal Diagnostic Center Nursing and Key Laboratory of Birth Defects and Related Diseases of Women and Children, West China Second University Hospital of Sichuan University, Chengdu, China

**Keywords:** sepsis, IL-1β, sepsis-associated encephalopathy, cognitive impairment, microglia activate

## Abstract

Sepsis is a leading cause of death resulting from an uncontrolled inflammatory response to an infectious agent. Multiple organ injuries, including brain injuries, are common in sepsis. The underlying mechanism of sepsis-associated encephalopathy (SAE), which is associated with neuroinflammation, is not yet fully understood. Recent studies suggest that the release of interleukin-1β (IL-1β) following activation of microglial cells plays a crucial role in the development of long-lasting neuroinflammation after the initial sepsis episode. This review provides a comprehensive analysis of the recent literature on the molecular signaling pathways involved in microglial cell activation and interleukin-1β release. It also explores the physiological and pathophysiological role of IL-1β in cognitive function, with a particular focus on its contribution to long-lasting neuroinflammation after sepsis. The findings from this review may assist healthcare providers in developing novel interventions against SAE.

## 1 Introduction

Sepsis is one of the leading causes of death worldwide and is characterized by multiple organ injuries resulting from dysregulated host response to infections (Liu et al., 2014; Singer et al., 2016). In addition to its high mortality rate, sepsis can also cause to long-lasting, even permanent, organ damages in septic survivors. It has been estimated that up to 40% of sepsis patients experience neurological and/or cognitive complications even after being discharged (Rudd et al., 2020; Fleischmann-Struzek et al., 2021; Sonneville et al., 2023). Some researchers prefer these prolonged and disabling forms of cognitive impairment in sepsis survivors as sepsis-associated encephalopathy (SAE) (Catarina et al., 2021). Although the exact underlying mechanism is not yet fully understood, it is widely accepted that neuroinflammation plays a significant role (Annane and Sharshar, 2015; Rengel et al., 2019; Pan et al., 2022; Sekino et al., 2022). During the initial systemic inflammation of sepsis, a large amount of cytokines are released, which could enter the central nervous system (CNS) via circumventricular organ and choroid plexus devoid of a blood–brain barrier (Shimada and Hasegawa-Ishii, 2017). Additional, systemic inflammation can also disrupt the blood–brain barriers (Gao and Hernandes, 2021; Sekino et al., 2022; Zou et al., 2022). Once inside the CNS, these cytokines activate microglia, the innate immune cells in the brain, to produce more pro-inflammatory cytokines such as IL-1β, tumor necrosis factor-α (TNF-α), and interferon-γ (INF-γ) (Hoogland et al., 2015; Zrzavy et al., 2019; Hu et al., 2023), ultimately resulting in long-lasting neuroinflammation that leads to neuron loss and cognitive impairment (Sekino et al., 2022; Hu et al., 2023). Among these pro-inflammatory cytokines, IL-1β is particularly important within the CNS after sepsis (Wang et al., 2012; Pozzi et al., 2018). Previous studies have shown that IL-1β, but not TNF-α or IL-6, is the exclusive cytokine within the CNS that has been verified to remain elevated for up to 70 days in rats survived neonatal sepsis but subsequently developed cognitive impairment (Lan et al., 2015; Liao et al., 2023). Furthermore, neurodevelopment of neonatal septic rats could be improved by directly antagonizing IL-1β (Zhang et al., 2022). This review summarizes recent advances in microglia activation-induced excessive IL-1β release and the molecular signaling pathways involved in IL-1β-induced cognitive dysfunction among sepsis survivors.

## 2 Activation of microglia during sepsis

As the primary immune cell within the CNS, microglia are activated by the recognition of danger signals such as invading pathogens and cytokines during systemic inflammation. For instance, inflammatory cytokines like TNF-α, IL-1β and IL-6 activate microglia through their specific receptors (van Gool et al., 2010).

**Figure 1:**
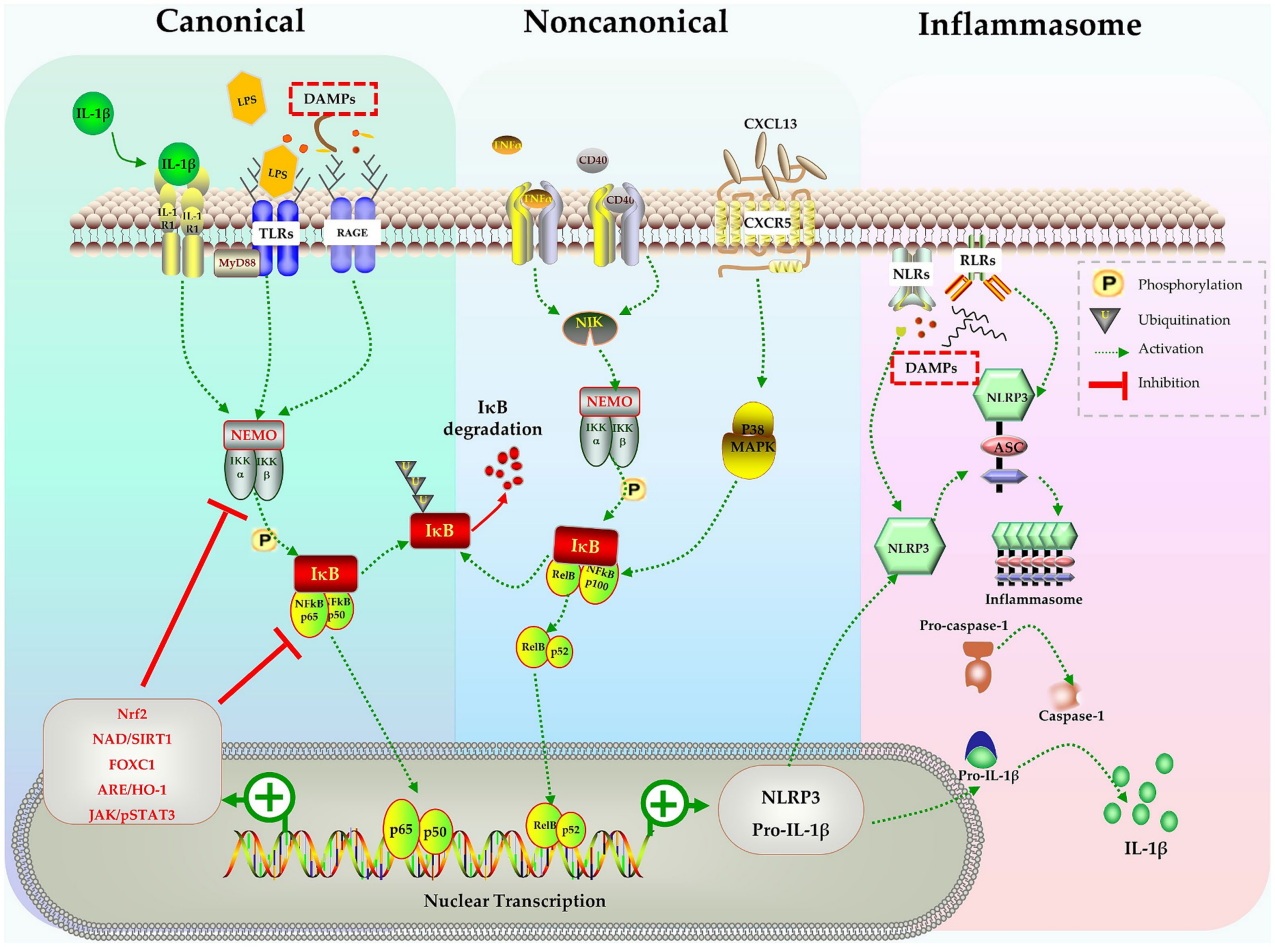
The NF-κB pathway in activation of microglia, inflammasomes formation and production of IL-1β. Stimulates like IL-1β, LPS, DAMPs trigger the canonical pathway, TNF-α and CD40 activate the noncanonical pathway, both pathways activate the NF-κB complex and causes nuclear transcription of NLRP3 and pro-IL-1β. NLRP3 assembles into inflammasome and activates caspase-1, finally leads to release of mature IL-1β. Many molecules in various signaling pathways can negatively regulate the NF-B pathway and inhibit the activation of microglia.

The nuclear factor kappa B (NF-κB) signaling pathway is a key signal pathway that modulates inflammatory responses. Studies have shown that the NF-κB signaling pathway is involved in the activation of microglia during sepsis (Mulero et al., 2019; Zhao et al., 2019; Nguyen et al., 2021; Wang H. et al., 2022). The NF-κB family consists of subunits p105/p50, p100/p52, p65/RelA, RelB, and c-Rel. These subunits reside in cytoplasm and bind with inhibitory IκB proteins to form an inactive complex (Mitchell et al., 2016; Mulero et al., 2019). The NF-κB signal can be activated through the canonical pathway or the noncanonical pathway. In the canonical pathway, inflammatory stimulation activates a complex containing NF-κB essential modulator (NEMO) and IκB kinases (IKK1/2). This complex phosphorylates IκB proteins that are bound to NF-κB in the cytoplasm, leading to the proteasomal degradation of IκBs. With the rapid degradation of IκBs, the p65:p50 heterodimer is released and translocates into nucleus, where it binds to κB site-containing DNA to enhance the transcription of target genes (Mitchell et al., 2016; Mulero et al., 2019). In the noncanonical pathway, signals such as the activation of TNF receptors by TNF-α or CD40 ligand, activate the NF-κB-inducing kinase (NIK) complex, which is identified as a mitogen-activated protein kinase (MAPK) kinase (Coope et al., 2002; Razani et al., 2011). NIK activates IKK1 through phosphorylation at Ser and Thr residues (Ling et al., 1998; Razani et al., 2011). The activated NIK/IKK1 complex phosphorylates inhibitor IκB and leads to the processing of precursor forms of NF-κB family members, particularly p100. p100 triggers IκB degradation and the release of the active p52 subunit, which forms active RelB:p52 heterodimers. These heterodimers then translocate into the nucleus and regulate specific gene transcription for immune responses (Ling et al., 1998; Coope et al., 2002; Mulero et al., 2019) (Figure 1).

Considering the crucial role of the NF-κB signaling pathway in microglia activation, numerous studies have indeed explored the potential of attenuating microglia activation by targeting the NF-κB signaling pathway. Zhao et al. demonstrated in mice that lipopolysaccharide (LPS) induced cognitive impairment and neuroinflammation occurred due to microglia activation via activation of the NF-kB signaling pathway. By pharmacological suppressing TLR-4/MyD88, they were able to abolish LPS-induced NF-κB activation and the release of pro-inflammatory cytokines, which in turn mitigated cognitive impairment (Zhao et al., 2019).

The nuclear erythroid 2-related factor 2 (Nrf2) is a transcription factor, which, under normal homeostatic conditions, is expressed with Kelch-like ECH-associated protein 1 (Keap1) in the cytoplasm. When stimulated by hazardous signals like oxidative or inflammatory stress, Nrf2 is released from Keap1 and translocates into the nucleus, where it binds to antioxidant response elements (AREs). This leads to the transcription of ARE genes involved in cellular defense and antioxidant responses, ultimately promoting cellular adaptation and survival under stress conditions (Saha et al., 2020). NF-κB is one of the downstream targets of Nrf2 that has been extensively studied. Nrf2 restricts the degradation of IκBα, thereby indirectly inhibiting the nuclear translocation of NF-κB and blocking the activation of the NF-κB signaling pathway (Yerra et al., 2013; Chen et al., 2018; Saha et al., 2020; Yu et al., 2020). Previous studies have highlighted the neuroprotective role of Nrf2 in various conditions, such as traumatic and ischemic brain injury (Dong A. et al., 2018; Dong W. et al., 2018; Yang et al., 2018). Chen et al. further demonstrated thathydrogen gas improved neuronal injury or cognitive dysfunction in mice with SAE through the activation of the Nrf2 pathway (Chen et al., 2021).

Forkhead box C1 (Foxc1) is another transcription factor implicated in the defense processes against oxidative damage, inflammation, and apoptosis (Xia et al., 2019; Zhao et al., 2020). Wang et al. showed with a cecal ligation perforation (CLP) sepsis model that overexpression of Foxc1 suppressed microglia activation related to the NF-κB pathway by enhancing IκBα expression, stabilizing the IκBα/p65 complex and reducing transcription driven by p65. This ultimately resulted in improved cognitive function in mice with SAE (Wang H. et al., 2022).

The Janus kinase/signal transducer and activator of transcription (JAK/STAT) signaling system is an essential cascade involved in cell proliferation, differentiation, and immune modulation (Xin et al., 2020). Therefore, it is not surprising that JAK/STAT signaling pathway interacts with the NF-κB pathway, and these interactions regulate immune responses during sepsis (Cai et al., 2015). Persistently activated JAK/STAT signaling is associated with prolonged inflammation and organ damage observed in sepsis (Cai et al., 2015; Wu et al., 2020; Xin et al., 2020). The complex interactions with other signaling pathways make the biological effects of JAK/STAT signaling more intricate. Septic cytokine-mediated activation of JAK/STAT3 signaling enhances STAT3 binding to the telomerase reverse transcriptase (TERT) promoter and leads to increased TERT protein expression (Cai et al., 2015; Wu et al., 2020). TERT, the telomerase protein, provides neuroprotection against LPS-induced brain injury by converting microglial polarization from M1 to M2 phenotype (Xin et al., 2020; Zhou et al., 2021). Furthermore, JAK2/STAT3 also modulates inflammatory responses through the α7 nicotinic receptor (α7nAChR), suppressing NF-κB and cytokine production and improving survival in sepsis (Peña et al., 2010; Zhang et al., 2017).

The chemokine C-X-C motif ligand 13 (CXCL13) and its receptor CXCR5 also play important roles in various immune and inflammatory processes (Kazanietz et al., 2019). Activation of the CXCL13/CXCR5 axis in microglia results in p38MAPK/NF-κB signal activation and promotes microglial polarization into the M1 phenotype. Conversely, down-regulation of CXCR5 suppresses neuroinflammation caused by microglial activation and alleviates cognitive dysfunction in a mouse model of sepsis (Li et al., 2017; Shen et al., 2021).

Silent information regulator 1 (SIRT1), a deacetylase-dependent nicotinamide adenine dinucleotide (NAD), is a key regulator in energy metabolism and tissue survival (Houtkooper et al., 2012; Jiao and Gong, 2020). The SIRT1 protein, which mediates oxidative respiration and anti-inflammatory responses, directly inhibits NF-κB signaling and mitigates SAE (Kauppinen et al., 2013). Specifically, SIRT1 deacetylates the RelA/p65 complex at Lys310, inhibits the nuclear translocation of NF-κB, and leads to IκBα-dependent nuclear export of NF-κB (Yeung et al., 2004; Han et al., 2019; Tian et al., 2019; Yang et al., 2020).

These studies have revealed the critical role of the NF-κB signaling pathway in microglial activation, neuroinflammation and cognitive impairment following sepsis. Moreover, they suggest that targeting the NF-κB signaling may be a potential intervention for improving cognitive dysfunction induced by sepsis (Figure 1).

In summary, the activation of NF-κB plays a pivotal role in initiating the transcription of pro-inflammatory cytokines, such as IL-1β. This NF-κB pathway is a component of a larger inflammatory response encompassing the activation of other signaling pathways and the release of inflammatory mediators. It is imperative to comprehend the molecular events that connect NF-κB activation and to the release of IL-1β in order to understand the mechanisms that drive inflammation and to devise therapeutic strategies to manipulating these processes in diverse diseases.

## 3 M1 microglia as the primary source of brain IL-1β during sepsis

Microglia, the main type of resident macrophages in the CNS, play a crucial role in immunological surveillance. Equipped with an array of pattern recognition receptors (PRRs), they detect changes in cytokine and chemokine levels as well as identify infections and damages (Ransohoff and Perry, 2009; Cherry et al., 2014; Roh and Sohn, 2018; Wesselingh et al., 2019). Therefore, it is not surprising that microglia can be activated by septic cytokine storms reaching the brain, utilizing the aforementioned pathways and receptors (Yan et al., 2022). Activation of microglia has been observed as early as 6 h following sepsis induced by CLP (Ye et al., 2019). Prolonged activation of microglia following sepsis has been associated with SAE. Wang et al. and Lan KM et al. discovered that following exposure to LPS in newborn rats resulted in sustained activation of microglia until postnatal day 71, leading to severe cognitive dysfunction (Wang et al., 2013; Lan et al., 2015). Numerous studies have indicated that inflammation caused by activated microglia is the main contributor to cognitive impairment following sepsis (Goshen et al., 2007; Ye et al., 2019; Zrzavy et al., 2019; Yan et al., 2022; Hu et al., 2023; Mordelt and de Witte, 2023). Neuroprotective benefits and reduced cognitive impairment induced by sepsis or surgery have been observed when microglia activation is prevented using the antibiotic minocycline (Michels et al., 2015; Tan et al., 2023). Unfortunately, minocycline failed to prevent postoperative cognitive dysfunction in a recent clinical trial, suggesting that the mechanisms underlying neuroinflammation-related brain dysfunction extend well beyond microglial activation (Takazawa et al., 2023).

Even in the context of microglial activation, there are two distinct phenotypes that can occur. Microglia can be activated and polarized into the M1 phenotype through the pathogen associated molecular patterns (PAMPs) and damage associated molecular patterns (DAMPs), which occurs via PRRs such as membrane Toll-like receptors (TLRs), Scavenger receptors (RAGE) and cytoplasmic Nod-like receptors (NLRs) and RIG-like receptors (RLRs) (Boche et al., 2013; Roh and Sohn, 2018; Hu et al., 2023). The M1 phenotype of microglia releases large amount of pro-inflammatory cytokines, including IL-1β, which induces inflammation to combat invading pathogens (Cherry et al., 2014; Wang et al., 2023). Morphologically, M1 phenotype microglia is characterized by larger amoeboid-like somata, with expression of Iba-1, CD68 and CD11b as the surface markers (Hoogland et al., 2015; Orihuela et al., 2016). Another activated phenotype of microglia is the M2 phenotype, which releases anti-inflammatory mediators and contributes to the resolution of inflammatory responses and tissue repair. The M2 phenotype microglia have a rod-like shape and express CD206 as a surface marker (Orihuela et al., 2016; Zhuang et al., 2020). Microglia play a crucial role in maintaining brain homeostasis through the balanced regulation of M1 and M2 phenotypes, as well as regulating immune response within the CNS. An imbalance in the M1/M2 ratio can have significant implications for neuroinflammation following sepsis (Orihuela et al., 2016; Hu et al., 2023; Wang et al., 2023). As demonstrated in previous studies, excessive production of IL-1β following M1 activation may lead to sustained neuroinflammation, contributing to the progression of cognitive dysfunction in neonatal sepsis. Conversely, an overactive M2 response may suppress necessary immune responses, impairing the ability to eliminate pathogens or clear cellular debris.

## 4 Activation of inflammasome and release of IL-1β

Inflammasomes are multiprotein complex modulating the inflammatory response and host defense against pathogens (Broz and Dixit, 2016). The inflammasome comprises several subunits, including nucleotide-binding oligomerization domain leucine-rich-repeat-containing pyrin 3 (NLRP3), the adaptor apoptosis-associated speck-like protein containing a caspase recruitment domain (ASC), and the effector molecule pro-caspase-1 (Paerewijck and Lamkanfi, 2022). Inflammasomes are assembled when microglia are stimulated by a variety of stimuli, such as PAMPs (viral, bacterial, or fungal pathogens) and DAMPs (extracellular ATP, mtDNA, ROS, etc.) (Broz and Dixit, 2016; Paerewijck and Lamkanfi, 2022). Upon autoproteolytic activation inside the complex, pro-caspase-1 is hydrolyzed into active caspase-1, which then processes pro-IL-1β and pro-IL-18 into their mature, active forms (Broz and Dixit, 2016; Paerewijck and Lamkanfi, 2022) (Figure 1). Active caspase-1 also cleaves gasdermin D (GSDMD) and triggers pyroptosis, resulting in release of mature IL-1β (Shi et al., 2017). It has been found that activated microglia, not astrocytes, are the sole source of NLRP3 inflammasome in CNS of rodents (Gustin et al., 2015; Moraes et al., 2023). Numerous studies have demonstrated that NLRP3-induced IL-1β release is the main cause of the brain dysfunction following sepsis (Sui et al., 2016; Xie et al., 2020; Yan et al., 2022). Pharmacological inhibition of NLRP3 not only reduces the expression of IL-1β, but also alleviates cognition impairment after sepsis (Sui et al., 2016; Xie et al., 2020; Li et al., 2022; Sekino et al., 2022; Moraes et al., 2023).

## 5 Effect of IL-1β on learning and memory

As a pro-inflammatory cytokine, IL-1β is involved in inflammation and the host’s defensive response. Once it binds with its receptor (IL-1R), IL-1β can increase the expression of adhesion factors and facilitate the chemotaxis of leukocytes to infection sites, intensifying the cascade of immune reaction (Weber et al., 2010). Aside from regulating immune responses, IL-1β in the CNS is also associated with learning and memory. The hippocampus is a critical structure located in the medial temporal lobe of the brain, playing a central role in learning and memory formation. It is particularly involved in the consolidation of short-term memory into long-term memory, as well as spatial memory and navigation. Both IL-1β and IL-1R are expressed in the hippocampus under normal physiological conditions (Huang and Sheng, 2010). Previous studies have demonstrated that a physiological level of IL-1β is necessary for memory formation, especially for hippocampus-dependent memory (Goshen et al., 2007; Yirmiya and Goshen, 2010; Wang et al., 2012). Disruption of IL-1 signaling through either the use of IL-1R antagonist (IL-1ra) or genetic deletion of IL-1R (IL-1rKO) impairs hippocampus-dependent memory (Avital et al., 2003; Goshen et al., 2007). Additionally, a physiological level of IL-1β is also essential for high-frequency stimulation-induced long-term potentiation (LTP) and normal hippocampal development (Goshen et al., 2007). Treatment with IL-1rKO or IL-1ra can impair memory formation and the maintenance of LTP (Schneider et al., 1998; Weber et al., 2010). On the contrary, excessive IL-1β produced detrimental effect on hippocampus-dependent memory formation and LTP maintenance. However, this effect can be reversed by blocking IL-1β signaling with IL-1ra, which improves cognitive function (Goshen et al., 2007; Weber et al., 2010). In summary, current data suggests that the effect of IL-1β on memory follows a U-shaped pattern: physiological level of IL-1β is necessary for memory formation and a slight increase in IL-1β can improve memory function. However, deviations from physiological range of IL-1β levels, whether through inhibition of IL-1β signaling or excessive IL-1β expression, lead to impaired memory formation and consolidation (Goshen et al., 2007; Weber et al., 2010).

## 6 IL-1β impairs cognitive function after sepsis

In addition to serving as pivotal pro-inflammatory cytokine in the innate immune response, IL-1β also plays a critical role in the pathogenesis of a variety of CNS diseases associated with cognitive impairment, such as epilepsy, stroke, schizophrenia, and autism (Iori et al., 2016; Krakowiak et al., 2017; Tsai, 2017). Research has provided evidence demonstrating that elevated expression of IL-1β can impair synaptic formation and function, thereby interfering normal neuronal communication and disrupting cognitive function.

### 6.1 Synapse formation

Synapse formation and maintenance encompass a series of sequential events that commence with the differentiation of neural precursor cells, followed by axonal migration and guidance, axonal and dendritic branch formation, and the maturation of synaptic circuits (Taverna et al., 2014; Harris and Littleton, 2015; Kay, 2016; Ulloa et al., 2018). At each stage, an elevated level of IL-1β has the potential to disrupt the establishment of functional synapses.

#### 6.1.1 Differentiation of neural precursor cell

Neurogenesis plays a crucial role in memory function. Neural precursor cells (NPCs) can differentiate into neurons, astrocytes, and oligodendrocytes (Lazutkin et al., 2019). Sustained expression of IL-1β inhibits neurogenesis and triggers apoptosis in NPCs (Wang et al., 2007; Lin Q. et al., 2019). IL-1β activates stress-activated protein kinase/c-Jun N-terminal kinase (SAPK/JNK), which phosphorylates and cleaves the Notch receptor. This results in the release and translocation of the Notch intracellular domain (NICD) into nucleus (Lin Q. et al., 2019). Once NICD binds to DNA, it promotes the expression of Hes1 and Hes5 genes, which induce NPCs to differentiate into glial cells. At the same time, IL-1β suppresses the expression of Mash1 and Ngn1 genes, thereby inhibiting NPCs from differentiating into neurons (Kageyama et al., 2008; Teng et al., 2009; Lin Q. et al., 2019). By inhibiting the IL-1β or SAPK/JNK signaling pathway, neurogenesis in NPCs can be restored and excessive gliogenesis can be prevented (Wang et al., 2007; Lin Q. et al., 2019).

#### 6.1.2 Synapse structure

To establish a sophisticated network, neurons extend their axonal and dendritic processes to connect with other neurons. Once inter-neuron contact is established, neurons undergo structural change to form new synapse. Synaptophysin (SYN) and post-synaptic density-95 (PSD-95), located in the pre-and post-synaptic membranes respectively, are commonly used molecular markers for evaluating synapse morphology (Dore and Malinow, 2021; Zhang and Augustine, 2021). Studies have demonstrated that microglia regulated formation and development of synapses. Stimulation with LPS increases the release of IL-1β and reduces production of IL-10 and TGF-α in activated microglia, leading to a decrease in the number of synapses between neurons as measured by SYN/PSD-95 colocalized puncta (Mishra et al., 2012; Lim et al., 2013; Hu et al., 2023). Additionally, excessive IL-1β suppresses the function of interleukin-1 receptor accessory protein like 1 (IL1RAPL1), which normally activates JNK and phosphorylates PSD-95. Suppressed IL1RAPL1 results in mis-location of PSD-95 in post-synaptic membranes and impair synaptic function (Pavlowsky et al., 2010; Pozzi et al., 2018).

Oligodendrocytes wrap around the axon to form myelinated axons and Ranvier nodes, increasing axonal conduction velocity. The differentiation and maturation of oligodendrocyte progenitor cells (OPCs) are essential for axonal remyelination. IL-1β also inhibits the normal differentiation of OPCs in postnatal sepsis rats (Ohtomo et al., 2018). Myelin basic protein (MBP), 2′, 3′-cyclic-nucleotide 3-phosphodiesterase (CNPase), and proteolipid (PLP) are well-recognized protein markers for mature myelination, and their expression can be significantly suppressed by IL-1β, resulting in axonal hypomyelination (Xie et al., 2016; Zhou et al., 2021). Furthermore, neurofilament proteins neurofilament-68(NFL), neurofilament-160(NFM), and neurofilament-200(NFH) play a vital role in providing structural support to neurons, as well as determining axonal diameter and conduction velocity. IL-1β can inhibit these neurofilament proteins by activating the p38MAPK pathway and suppressing FYN/MEK/ERK phosphorylation (Xie et al., 2016; Zhou et al., 2021). This leads to hypomyelination, destruction of myelin sheaths, and damage to Ranvier nodes, resulting in small axon diameter and abnormal axon morphology (Wang et al., 2013; Xie et al., 2016; Han et al., 2017; Zhou et al., 2021).

**Figure 2:**
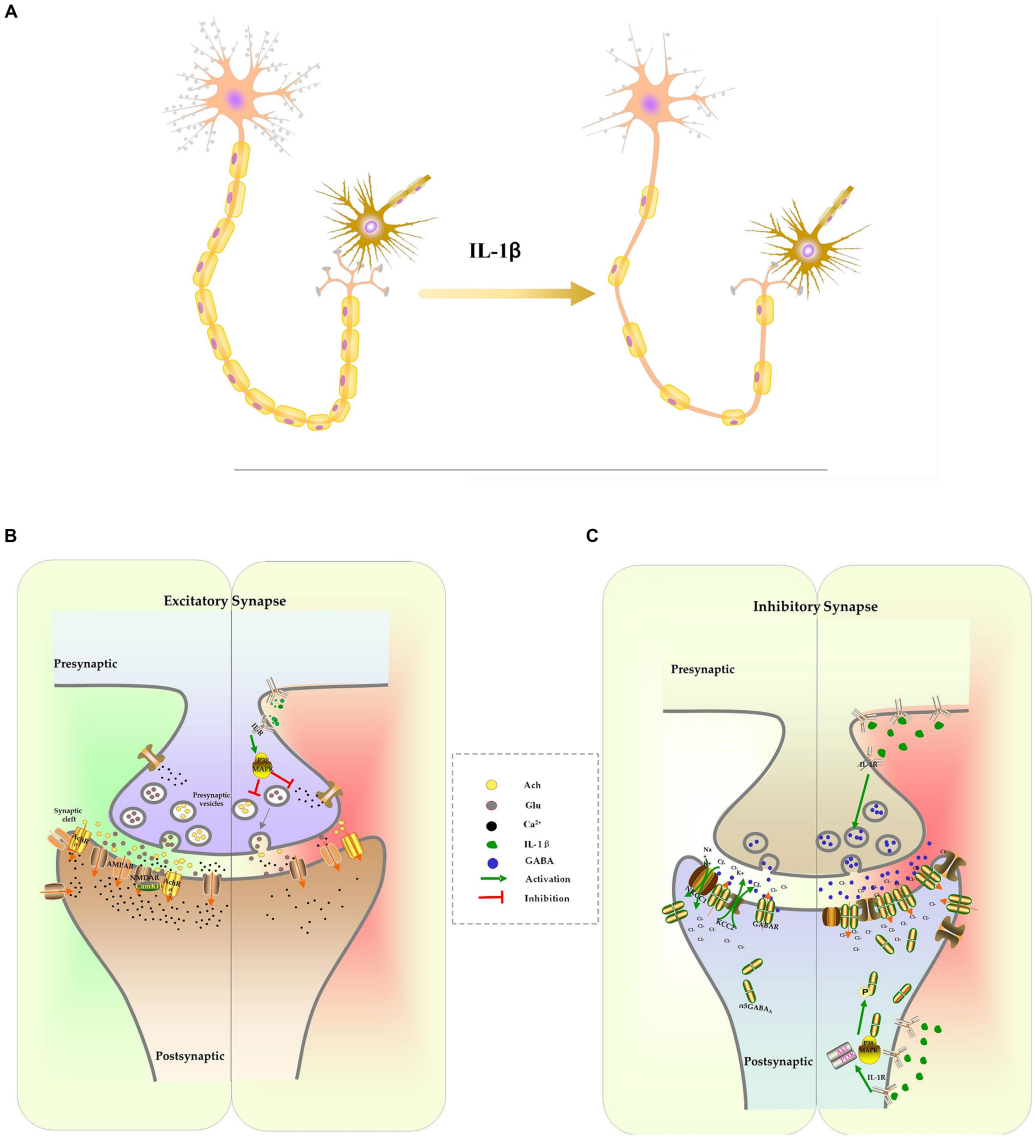
The effect of IL-1β on neuron development and synapse formation. Excessive IL-1β suppresses the formation of dendrites and dendritic spines, which results in a reduction of the complexity of dendrites and the density of spines. It also inhibits the expression of myelination protein, resulting in axonal hypomyelination **(A)**. IL-1β suppresses expression of receptors and release of excitatory neurotransmitters, culminating in inhibition of the glutamatergic system and cholinergic system **(B)**. IL-1β increases the expression of the GABA receptor and the release of GABA. IL-1β interferes with chloride homeostasis by changing NKCC1/KCC2, which delays the E/I switch **(C)**.

In addition, IL-1β inhibits the formation of dendrites and dendritic spines. Sustained elevations of IL-1β in the hippocampus lead to a decrease in the complexity of dendritic trees, including reduced branch length, dendritic spine density, and dendritic branch density (Liu et al., 2018; Lin L. et al., 2019). Methyl CpG binding protein 2 (MeCP2) is a transcriptional regulator involved in controlling spine morphogenesis and plasticity. IL-1β-induced up-regulation of MeCP2, through the activation of the PI3K/AKT/mTOR signaling pathway, decreases the expression of PSD-95 and spine density (Chao et al., 2007; Cheng and Qiu, 2014; Tomasoni et al., 2017; Pozzi et al., 2018). Brain-derived neurotrophic factor (BDNF), a member of the neurotrophin family, is known to play a vital role in plasticity, neuronal survival, synaptic development, dendritic branching, and long-term potentiation (LTP) (Wang C. S. et al., 2022). When BDNF binds to its high-affinity receptor TrkB, it activates cAMP Response Element-Binding Protein (CREB) via the PI3K/Akt signaling pathway and promotes the transcription of Arc and Homer1. Arc and Homer1 facilitate the organization of filamentous actin (F-actin) and remodeling of spines, which are crucial processes for structural alterations of spines and stabilization of the potentiation effect (Tong et al., 2012; Giacobbo et al., 2019; Eriksen and Bramham, 2022). IL-1β disrupts cytoskeletal alterations and reduces the density of mature spines through the aforementioned p38MAPK signaling pathway (Tong et al., 2012; Herrera et al., 2019) (Figure 2A).

### 6.2 Neurotransmitter and receptor

#### 6.2.1 Glutamatergic system

Glutamate is one of the excitatory neurotransmitters in CNS. It is primarily synthesized in glial cells as glutamine and transported into neurons, where it is converted into glutamate by glutaminase, and stored in secretory vesicles. Following stimulation, the vesicles merge with the cellular membrane, allowing glutamate to be released into the synaptic space (Marmiroli and Cavaletti, 2012). Once it binds to its receptors, primarily the N-methyl-D-aspartate receptor (NMDAR) or the alpha-amino-3-hydroxy-5-methylisoxazole-4-propinonic acid receptor (AMPAR), glutamate induces calcium influx, activates intracellular kinases and phosphatases, and facilitate synaptic transmission. These processes are involved in various neuronal activities, including neuronal maturation, synaptogenesis, learning, and memory (Marmiroli and Cavaletti, 2012). The suppression of the glutamatergic system has been associated with cognitive dysfunction. Pro-inflammatory cytokine IL-1β inhibits glutamate release in presynaptic terminals and impairs memory consolidation by activating p38MAPK, inhibiting ERK phosphorylation and calcium influx (Kelly et al., 2003; Gonzalez et al., 2013; Machado et al., 2015; Chakraborty et al., 2023). It has been observed that excessive IL-1β also suppresses the function and expression of glutamatergic receptors in sepsis models. The reconsolidation of memories following contextual fear memory reactivation was impaired as IL-1β reduces surface expression of AMPA receptor subunit GluA1 and decreased its phosphorylation at Serine 831/845 (Machado et al., 2018). Additionally, IL-1β inhibits the expression of the NR2A and NR2B NMDAR subunits. This effect can be reversed by inhibiting IL-1β signaling with IL-1ra or by activating NMDAR with an agonist (Di Filippo et al., 2013; Taoro-González et al., 2019) (Figure 2B).

Other studies have shown that IL-1β can produce opposite effects on the glutamatergic system in different CNS diseases and chronic pain (Dong et al., 2017; Qiu et al., 2020; Choi et al., 2021). Activated microglia and excessive IL-1β can enhance glutamate release, increase NMDAR expression, and result in significant calcium inflow, leading to excitotoxicity and oxidative stress damage (Mishra et al., 2012; Dong et al., 2017; Qiu et al., 2020). To accurately determine the impact of IL-1β on the glutamatergic system in various neuroinflammatory diseases, more detailed and disease-specific research is required.

#### 6.2.2 Cholinergic system

Another crucial system involved in cognitive functions is the cholinergic signaling system. In neurons, the choline acetyltransferase enzyme converts acetyl-CoA and choline into acetylcholine (ACh). ACh is stored in vesicles and released into the synaptic cleft in response to specific stimuli. ACh activates heterotrimeric G proteins, resulting in the gating of cation channels and the activation of downstream signaling pathway through binding to muscarinic (mAChRs) or nicotinic ACh receptors (nAChRs) (Maurer and Williams, 2017). ACh is degraded by the enzyme acetylcholinesterase (AChE) into choline and acetic acid and then re-uptaken by neurons. Studies have demonstrated that cholinergic transmission through M1-type mAChRs and 7nAChRs activates calcium influx, leading to increased stability of F-actin and dendritic spines, thereby modifying synaptic plasticity and stabilizing LTP (Maurer and Williams, 2017; Solari and Hangya, 2018). Elevated levels of IL-1β in the CNS impair cognition by upregulating AChE expression and activity, as well as suppressing ACh synthesis and release (Main et al., 1993; Li et al., 2000; Zhang et al., 2014). Inhibiting AChE can raise ACh levels in the CNS, thereby improving cognitive function in mice exposed to LPS (Liu et al., 2018). The cholinergic anti-inflammatory pathway, which is activated by increased ACh binding to 7nAChR, activates both STAT3/Jak2 and Nrf2 signaling pathways to prevent NF-κB-induced release of proinflammatory cytokines. This pathway is another mechanism responsible for the observed protective effect of AChE inhibitors on cognitive function (Peña et al., 2010; Zhang et al., 2017; Liu et al., 2018; Benfante et al., 2021) (Figure 2B).

#### 6.2.3 GABAergic system

Gamma-aminobutyric acid (GABA) is the principal inhibitory neurotransmitter in the brain. Glutamate is converted to GABA by the enzyme glutamic acid decarboxylase. GABA is then converted to succinic semialdehyde by GABA-transaminase, which is ultimately converted to succinate by the enzyme succinic semialdehyde dehydrogenase (Sears and Hewett, 2021). GABA is released into the synaptic cleft from presynaptic vesicles and binds to postsynaptic receptors allowing chloride to enter the neuron and causing postsynaptic hyperpolarization. The GABAergic system plays a significant role in regulating synaptic plasticity, learning, and memory (Sakimoto et al., 2021; Sears and Hewett, 2021). Dysfunction of the GABAergic system, particularly with reduced GABA system function, leads to cognitive impairment (Prévot and Sibille, 2021). In the hippocampal CA1 subfield, activation of the alpha5 subtype of GABA_A_ receptors (alpha5GABAA) primarily produces tonic inhibitory conductance (Sakimoto et al., 2021). Increased alpha5GABAA activity impairs memory performance (Martin et al., 2009, 2010).

IL-1β increases GABA levels in the brain by inhibiting reuptake and enhancing GABA release from activated glial cells (Wu et al., 2007; Lee et al., 2010). Additionally, IL-1β upregulates the expression of alpha5GABAA receptors on the surface of proximal dendrites. Activation of IL-1β leads to phosphorylation of the 2/3 subunits of GABAA receptors at Ser-408-409 through the p38MAPK and PI3K-Akt signaling pathways, resulting in the translocation of intracellular receptors to the neuronal membrane, and an increase in tonic inhibitory conductance (Serantes et al., 2006; Luscher et al., 2011; Wang et al., 2012; Prévot and Sibille, 2021).

IL-1β also disrupts the excitatory-to-inhibitory GABA switch during early CNS development in the context of neonatal sepsis. The balance between the chloride importer Na^+^-K^+^-2Cl-cotransporter (NKCC1) and the chloride exporter K^+^-Cl^−^ cotransporter (KCC1) determines the direction of chloride ion flow across the cellular membrane, which in turn determines the depolarization or hyperpolarization of the neuron following GABA stimulation. In the early postnatal stage, NKCC1 is the primary chloride transporter, leading to the accumulation of high chloride concentration inside the neuron and mediating the depolarizing effects of GABA (Peerboom et al., 2023). As the CNS matures, KCC2 becomes the dominant chloride transporter, resulting in a decrease in intracellular chloride ion concentration. This causes chloride influx following GABA activation, which further hyperpolarizes the neuron (Peerboom et al., 2023). The excitatory-to-inhibitory (E/I) switch in GABA function during CNS development is essential for cell proliferation, differentiation, early network wiring, synapse development, and neural plasticity (Deidda et al., 2015a; Virtanen et al., 2021; Peerboom et al., 2023). Our recent research has shown that increased IL-1β in the hippocampus, caused by severe inflammation, upregulates KCC2 expression during early CNS development, accelerating the GABA E/I switch and causing long-lasting cognitive dysfunction. Specific knockdowns of IL-1β or KCC2 expression can reverse this severe inflammation-induced cognitive impairment (Zhang et al., 2022). Interestingly, others have reported the opposite effect of IL-1β on the GABA E/I switch, where overexpression of IL-1β following maternal immune activation (MIA) leads to increased expression of the NKCC1 transporter and delayed GABA E/I switch in offspring. The defected GABA E/I switch has been associated with CNS diseases such as epilepsy (Corradini et al., 2018). Furthermore, IL-1β-mediated E/I switch defects was also involved in other neurodevelopmental disorders, including Down syndrome and autism spectrum disorders (ASD) (Tyzio et al., 2014; Deidda et al., 2015b; Inui et al., 2015). Inhibiting IL-1β signaling can be improve cognitive impairment in certain disorders (Figure 2C).

### 6.3 LTP

Long-term potentiation (LTP) is a process where synaptic efficacy is enhanced following high-frequency stimulation. It is considered as the cellular foundation for memory and learning (Dringenberg, 2020). Excitatory neurotransmitters activate postsynaptic NMDAR, resulting in the influx of calcium into the postsynaptic compartment. This calcium influx triggers a series of events, including the translocation of actin and its regulators to the dendritic spine. The translocation of actin to the spine is followed by the translocation of AMPA receptors (AMPARs), actin-binding proteins, and kinases, ultimately leading to the expansion of the dendritic spine (Bosch et al., 2014). AMPARs are ionotropic transmembrane receptors for glutamate that facilitate rapid synaptic transmission in the central nervous system (Armstrong et al., 2006). The translocation of these components to the synapse is essential for synaptic plasticity (Hanley, 2018). Furthermore, an increase in postsynaptic Ca^2+^ concentration promotes Ca^2+^/calmodulin-dependent protein kinase II (CaMKII) to phosphorylate various substrates, including the transcription factors CREB, GluA, and GluN2A/2B, as well as the synaptic scaffolding protein Homer and PSD-95. These substrates can be phosphorylated to activate multiple downstream signaling pathways and expedite the synaptic consolidation process (Hayashi, 2021).

In the CNS, dendritic spines, which resemble tiny mushrooms-like protrusions on dendrites, generally serve as the sites for the formation of excitatory synapses (Bosch and Hayashi, 2012; Bosch et al., 2014). In summary, the formation and maintenance of LTP, a key mechanism in learning and memory, depend on the normal structure and function of synapses, particularly at the sites of dendritic spines in the CNS (Choi and Kaang, 2023). Therefore, it is unsurprising to find that excessive expression of IL-1β in CNS affects the development of the synapse’s axon, dendrite, and dendritic spine as well as its cholinergic, glutamatergic, and GABAergic neurotransmitter systems, thereby inhibiting long-term potentiation (LTP) and impairing cognition (Bosch et al., 2014; Hayashi, 2021). The specific mechanisms by which IL-1β affects LTP are not fully understood and should be investigated within a disease-specific context.

## 7 Feed-forward loop in neuroinflammation

The sustained elevation of IL-1β levels in the CNS after a single dose of LPS treatment raises another important and interesting question: what is the underlying mechanism for prolonged microglia activation and IL-1β release after transient systemic inflammation? In the context of neuroinflammation, researchers have identified a feed-forward loop between neurons and microglia (Terrando et al., 2010; Lan et al., 2015). They have discovered that the IL-1 receptor antagonist (IL-1ra) not only decreases IL-1β-induced long-lasting cognitive impairments but also suppresses microglia activation and IL-1β production (Terrando et al., 2010; Wang et al., 2013; Lan et al., 2015). In the absence of pathogen or PAMPs, microglia are activated by DAMPs, which are only produced by dying cells (Lehnardt, 2010; Ransohoff and Brown, 2012). Together, they have concluded that IL-1β-mediated neuroinflammation caused neuronal death, which then released DAMPs to activate microglia and release more IL-1β, creating a feed-forward loop that leads to chronic neuroinflammation and long-term cognitive dysfunction (Terrando et al., 2010; Wang et al., 2013; Lan et al., 2015).

**Figure 3:**
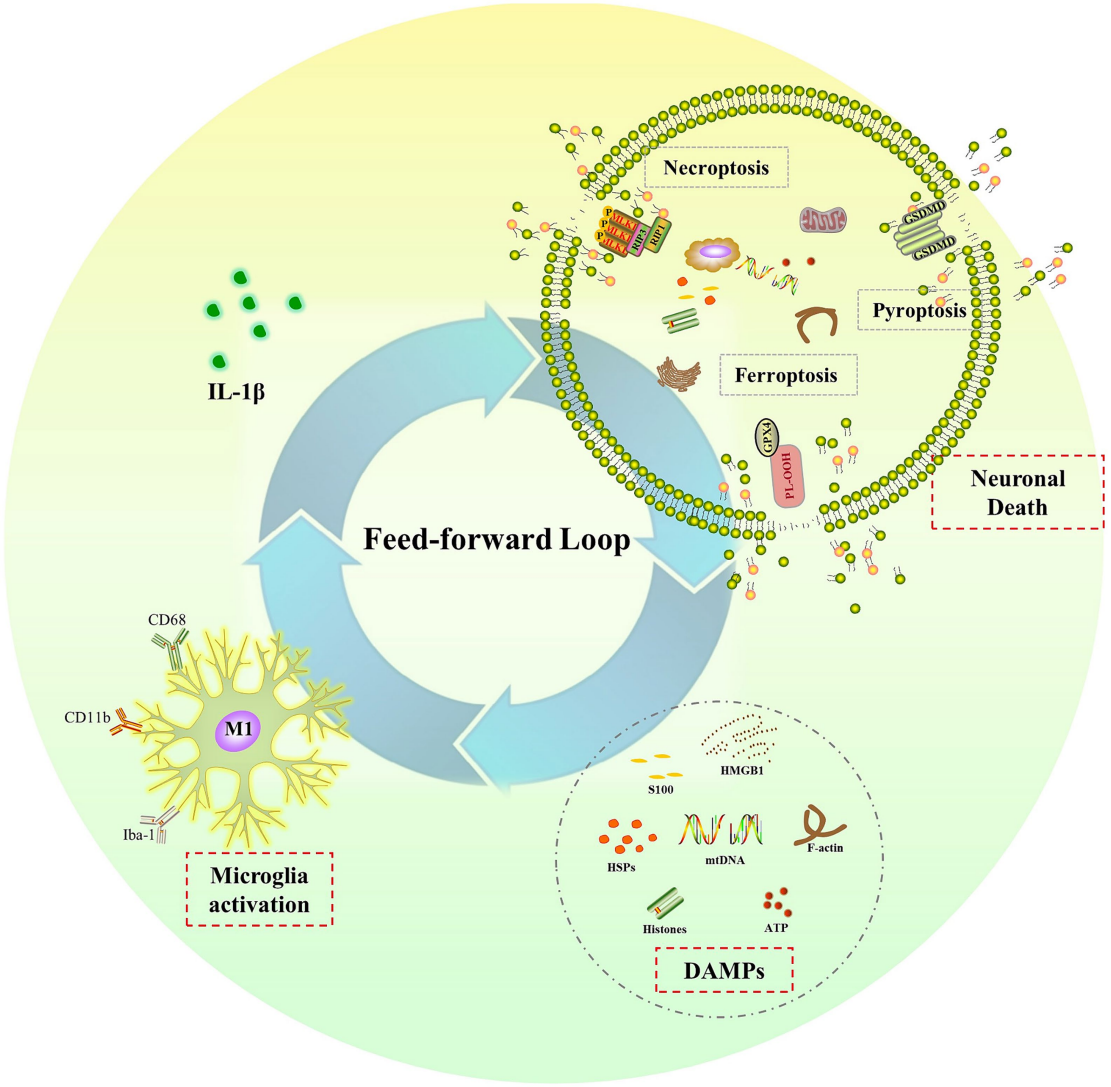
The feed-forward loop in chronic neuroinflammation. Elevated IL-1β induces neuronal death, such as necroptosis, pyroptosis and ferroptosis, leading to the release of DAMPs, which in turn activate microglia and result in more IL-1β release and chronic neuroinflammation.

Endogenous DAMPs are molecules that promote inflammation and passively leak out of dying cells when membranes rupture. Numerous cell death processes, including necrosis/necroptosis, pyroptosis, and ferroptosis, are connected to the release of DAMPs (Murao et al., 2021). All these different types of cell death have been reported in the pathological development of sepsis and cognitive dysfunction associated with sepsis (Fu et al., 2019; Zhou et al., 2019; Chu et al., 2022; Liao et al., 2022; Wang J. et al., 2022; Liao et al., 2023). Our recent research showed that necrostatin-1 and xenon were protective against sepsis-induced brain injury by preventing necroptosis. Necroptosis was activated via receptor interacting protein 1/3 (RIP1/3), which contributed to impaired neurodevelopment in neonatal sepsis survivors (Liao et al., 2022, 2023). The NLRP3/caspase-1 pathway-induced pyroptosis has also been linked to cognitive impairment following sepsis. Inhibiting pyroptosis by inhibiting NLRP3 and caspase-1 expression has shown protective effects on cognitive impairment following sepsis in newborn rats (Fu et al., 2019; Zhou et al., 2019). According to Wang (Wang J. et al., 2022) and Chu (Chu et al., 2022), ferroptosis contributed to cognitive dysfunction following SAE via the Nrf2/ferroptosis-related protein (GPX4) signaling pathway. In conclusion, all these types of cell death that release DAMPs may create a feed-forward loop that eventually results in chronic neuroinflammation and cognitive impairment after sepsis. To break the feed-forward cycle, future research should focus on the molecular mechanisms linking cell death and persistent neuroinflammation. It should also investigate possible therapeutic interventions for sepsis-related cognitive impairment (Figure 3).

## 8 Conclusion

Significant advancements have been made in the past two decades regarding our understanding of the correlation between neuroinflammation and post-septic cognitive impairment. This review encompasses an examination of the diverse effects of IL-1β generated following microglial activation on cognitive impairment, as well as delves into the underlying molecular mechanisms behind microglia activation, the influence of IL-1β on synapse development, and the impact of impairment on synaptic function. Furthermore, we explore the feed-forward loop mechanism, which relies on different types of cell death and releases endogenous DAMPs to activate the innate immune system and create persistent neuroinflammation. Researchers have conducted experimented utilizing several approaches based on these mechanisms, yielding encouraging results. Given the intricate interplay among these processes, a multifaceted approach should be employed in future preclinical and clinical trials to target various pathways in order to prevent or treat cognitive impairment following sepsis.

## Author contributions

QZ: Writing – original draft, Writing – review & editing. LW: Writing – original draft, Writing – review & editing. HH: Writing – original draft, Writing – review & editing. ZL: Writing – original draft, Writing – review & editing.
